# The efficacy of low-dose tadalafil in patients undergoing hemodialysis with end-stage renal disease

**DOI:** 10.1080/0886022X.2017.1349678

**Published:** 2017-07-25

**Authors:** Mustafa Suat Bolat, İsmail Özer, Onder Cinar, Ekrem Akdeniz, Ramazan Aşcı

**Affiliations:** a Department of Urology, Samsun Training and Research Hospital, Health Sciences University, Samsun, Turkey;; b Department of Nephrology, Samsun Training and Research Hospital, Health Sciences University, Samsun, Turkey;; c Department of Urology, Ondokuz Mayıs University, Samsun, Turkey

**Keywords:** End-stage renal disease, hemodialysis, sexual dysfunction, psychology

## Abstract

**Background:** Erectile dysfunction (ED) is a disorder that is frequently observed in people with chronic kidney disease who undergo hemodialysis (HD). In the context of evidence-based medicine, we aimed to investigate the effect of low-dose tadalafil on sexual function in patients undergoing HD.

**Methods:** The medical records of 30 males (aged 29–65 years) with end-stage renal disease (ESRD) on a HD program, and who had received 5 mg tadalafil twice weekly, were retrospectively evaluated. Changes in erectile and ejaculatory function were evaluated using the International Erectile Function Index questionnaire, the Erection Hardness Scale (EHS), and the Male Sexual Health Questionnaire (MSHQ).

**Results:** The mean age of the patients was 47.6 ± 10.1 years, their mean body mass index was 24.3 ± 4.2 kg/m^2^, their mean hemoglobin was 11.9 ± 0.9 g/dL, and their mean creatinine clearance was 5.8 ± 1.1 mL/min. At the third month of treatment, 36.6% of the patients had no ED, 40% had mild ED, 10% had mild-to-moderate ED, and 13.3% had moderate ED. The mean MSHQ scores (*p* < .05) and the mean EHS scores (*p* = .001) were significantly improved. There was no significant difference between Beck's Depression Inventory scores (*p* > .05), but Hamilton anxiety rate scores decreased significantly (*p* = .001). The quality-of-life score improved throughout the study period (*p* < .05).

**Conclusions:** Tadalafil therapy is an effective therapeutic option in patients with ESRD who undergo HD, not only for the treatment of ED, but also for ejaculatory function, with acceptable adverse effects.

## Introduction

The delicate balance between detumesence and erection is maintained by harmonious interaction of the sympathetic and parasympathetic nervous systems. Alfa-1 adrenergic stimulation, which is mediated by various mediators, leads to penile smooth-muscle contraction in the flaccid phase of the penis [1]. Parasympathetic stimulation results in neurogenic nitric oxide synthase (nNOS) expression, and nNOS uses molecular oxygen and L-arginine to produce nitric oxyde (NO). The NO that is produced then stimulates cyclic-GMP by protein kinase G, and finally, smooth-muscle relaxation occurs in the penis [2].

Erectile dysfunction (ED) is a disorder that is frequently observed with increasing prevalence in people with end-stage renal disease (ESRD) who undergo a hemodialysis (HD) program [3]. Several factors affect erectile function, such as low responsivenes of Leydig cells, inhibitors of Leydig cell receptors and endothelial dysfunction, which are necessary for libido, penile morphology, and penile function [4,5].

ESRD has become a global problem, and its incidence is increasing [6]. The chronic nature of the disease also leads to some other systemic problems, such as cardiovascular, skeletal, hematologic, neurogenic, immunologic and sexual disorders [7–10]. Although sexual problems are not life-threatening, they negatively affect the quality of life of people with ESRD when they occur [11].

Several studies have reported that ED prevalence is as high as 80% in ESRD people on an HD program than in the normal population [12–14]. In addition, in people with ESRD undergoing HD, sexual dysfunction occurs due to psychogenic, as well as organic, causes [15].

In the present study, we aimed to investigate the effect of 5 mg tadalafil, administered twice-weekly for three months, on sexual function in people with ESRD undergoing HD, using the International Index of Erectile Function questionnaire (IIEF) [16] and the Male Sexual Health Quality questionnaire (MSHQ) [17], in the context of evidence-based medicine.

## Materials and methods

This study was designed as a retrospective clinical trial. After obtaining approval from the Ethics Committee of Ondokuz Mayis University, Samsun, Turkey, all the patients were informed of the study for ethical reasons, and their written consents were obtained. The required number of patients was determined using a power analysis. The medical records of 30 males (aged 29–65 years) with ESRD and on HD who had previously consulted our clinic regarding sexual dysfunction, and had received 5 mg tadalafil twice weekly, were retrospectively evaluated [18]. At the beginning of the study, the patients included were assessed in terms of age, body mass index (BMI), urinary output volume (anuria, oliguria or normouria), hemoglobin, serum lipid profile (triglycerides and total cholesterol), hormonal status (total testosterone and prolactin) and creatinine clearance. A testosterone level of greater than 8.4 mmol/L and a prolactin level of less than 15 ng/mL was accepted as normal, and the diagnosis of ESRD was made by a nephrologist. Inclusion criteria were age over 18 years, an HD program for at least 6 months, and having a sexual partner, while exclusion criteria were previous urogenital surgery and cardiac comorbidity.

The questionnaires were interviewed face to face with patients in one session. As needed, quantitative variables were repeated throughout the study period. Changes in erectile function were evaluated using the short form of the Turkish validated IIEF [19], which consisted of six questions 1–5 and 15. Each question was scored from 1 (‘almost never’ or ‘never’) to 5 (‘almost always’ or ‘always’), and total scores were recorded. The patients were classified as having severe dysfunction (score = 0–6), moderate dysfunction (score = 7–12), mild-to-moderate dysfunction (score= 13–18), mild dysfunction (score = 19–24) and no dysfunction (score = 26–30). Erectile hardness quality was evaluated using the Erection Hardness Scale (EHS), consisting of four grades: the penis is larger but is not hard (Grade 1); hard, but not hard enough for penetration (Grade 2); hard enough for penetration, but not completely hard (Grade 3); and completely hard and fully rigid (Grade 4). Ejaculatory function was assessed using the short form of the Turkish validated MSHQ [20], consisting of four questions ranging from 0 to 5 scores. The first, second and third of these questions assess the behavior, frequency and power of the ejaculatory function (MSHQ 123) (scored from 0–15), and the fourth question assesses the ejaculation bother (MSHQ EjD) (scored from 0–5). The higher the sum of the first three MSHQ scores, the better the ejaculatory function; and the higher the last score, the better the ejaculatory function.

The psychological state of the patients was assessed as minimal, mild, moderate or severe depression, using the Beck’s Depression Inventory (BDI) [21,22] and the Hamilton Anxiety Rating Scale (HARS) [23,24]. The BDI consists of 21 questions, which are scored as follows: not at all-0; mild-1; moderate-2 and severe-3, respectively. A total score of 0–10 is considered normal, 11–16 = mild mood disturbance, 17–20 = borderline clinical depression, 21–30 = moderate depression, 31–40 = severe depression and ≥41 = extreme depression. The HARS consists of 17 questions, of which questions 1 and 3–9 are scored as not at all-0; moderate-1 and severe-3; and questions 2 and 10–17 s are scored from 0 (not at all) to 4 (severe). A score of less than 17 = mild anxiety, 18–24 = mild-to-moderate anxiety and >25 moderate-to-severe anxiety. Cronbach α reliability coefficiencies were 0.82 for IIEF; 0.79 for MSHQ, 0.87 for BDI and 0.76 for HARS.

The statistical analysis was carried out using Statistical Package for the Social Sciences for Windows software, version 21.0 (IBM, Chicago, IL) and the Shapiro–Wilk test was used to analyze the normal distribution of the quantitative data. The results are given as mean ± standard deviation, *n* (%) and/or median (min–max). The intragroup data were compared using the Wilcoxon signed rank test for non-normal data. Comparisons between data were performed using a paired-sample test. Pearson’s chi-square and Fisher’s exact test were used for comparisons of percentages. A *p* values of less than 0.05 was considered statistically significant.

## Results

The mean age of the patients was 47.6 ± 10.1 years, their mean BMI was 24.3 ± 4.2 kg/m^2^, and their mean hemoglobin was 11.9 ± 0.9 g/dL. The mean HD duration was 78.4 ± 27 months (24–120), the mean creatinine clearance was 5.8 ± 1.1 mL/min (4–8), and the frequency of HD was three sessions per week. The mean total serum testosterone level was 7.2 ± 1 (4.4–8.9) mmol/L, and only four patients were in the normal limits. The mean prolactin level was 5.9 ± 2.4 (3–12) ng/mL, and 11 patients were hypercholesterolemic and hypertriglyceridemic (36.6%) (Table 1).

**Table 1.** Demographic characteristics of the patients.

No. of the patients	30
Age (years), (mean ± SD)	47.6 ± 10.1
BMI^a^ (kg/m^2^)	24.3 ± 4.2
Hb^b^ (g/dL)	11.9 ± 0.9
Total cholesterol (mg/dL)	202.6 ± 6
Triglyceride (mg/dL)	190.3 ± 36.2
Total testosterone mmol/L	7.2 ± 1.0
Prolactine ng/mL	5.9 ± 2.4
Hemodialysis duration (months), (mean ± SD), (min–max)	78.4 ± 27 (24–120)
Hemodialysis frequency (*n*) (per week)	3
Creatinine clearence (mL/min), (mean ± SD), (min–max)	5.8 ± 1.1 (4–8)
Comorbidity (*n*, %)	
Diabetes mellitus	19 (63.3)
Hypertension	6 (20.0)
Polycystic kidney	1 (3.4)
Idiopathic	4 (13.3)

a BMI: body mass index.

b Hb: hemoglobin.

At the beginning of tadalafil treatment, 10 of the patients had mild ED (33.3%), six had mild-to-moderate ED (20%), and 14 had moderate ED (46.6%); at the first month of treatment, 15 patients had mild ED (50%), five had mild-to-moderate ED (16.6%), and eight had moderate ED (28.6%). At the third month of treatment, 11 patients had no ED (36.6%), 12 had mild ED (40%), three had mild-to-moderate (10%), and four had moderate ED (13.3%). Overall, the ED rate was 100%, 93.3% and 66.6% at pretreatment, at the first treatment month, and after the third month of treatment, respectively (Table 2).

**Table 2.** Erectile dysfunction classification of the patients.

	Pretreatment (*n*, %)	First month of treatment (*n*, %)	Third month of treatment (*n*, %)
Severe ED (0–6)	0	0	0
Moderate ED (7–12)	14 (46.6)	8 (28.6)	4 (13.3)
Mild to moderate ED (13–18)	6 (20.0)	5 (16.6)	3 (10.0)
Mild ED (19–25)	10 (33.3)	15 (50.0)	12 (40.0)
No ED (>26)	0	2 (6.6)	11 (36.6)
Overall ED	30 (100)	28 (93.4)	23 (66.6)

The mean IIEF scores at pretreatment and at the first and third month of treatment were 21 ± 4.7, 21.4 ± 4.0 and 22.6 ± 4.0, respectively. Although there was no statistically significance between the pretreatment and first month of treatment values (*p* = .05), the third month of treatment score was significantly increased compared to the pretreatment value (*p* = .001) (Table 3).

**Table 3.** Findings of sexual, psychogenic, and quality of life through tadalafil treatment.

Variable	Pretreatment	First month of treatment	Third month of treatment	Difference between pretreatment and third month (%)	*p*
IIEF^a^ (1–5,15)	21 ± 4.7	21.4 ± 4.0	22.6 ± 4.0	+7.6	Prefirst-mo. treatment .05 Prethird-mo treatment .001
MSHQ123^b^	9.9 ± 2.4	10.6 ± 2.6	11.4 ± 0.7	+14.1	Prefirst-mo. treatment .04 Prethird-mo treatment .001
MSHQ EjD^c^	3.1 ± 0.9	3.6 ± 0.7	4.0 ± 0.7	+31.7	Prefirst-mo. treatment .001 Prethird-mo treatment .001
EHS^d^	3.3 ± 1.1	3.6 ± 0.7	3.7 ± 0.7	+12.1	Prefirst-mo. treatment .001 Prethird-mo treatment .001
Beck’s score	2.9 ± 5.4	2.5 ± 2.2	2.9 ± 2.3	−8.8	Prefirst-mo. treatment .49 Prethird-mo treatment .32
Hamilton score	19.3 ± 4.2	12.5 ± 3.1	10.5 ± 2.6	−45.6	Prefirst-mo. treatment .001 Prethird-mo treatment .001
QoL^e^ score	3.4 ± 0.7	3.1 ± 0.5	2.8 ± 0.4	+17.6	Prefirst-mo. treatment .04 Prethird-mo treatment .02

a Short form of International Index of Erectile Function score.

b MSHQ 123 defines ejaculatory function.

c MSHQejD defines ejaculatory dysfunction.

d Erection Hardness Scale.

e QoL: quality of life score.

The mean MSHQ123 scores at pretreatment and at the first and third month of treatment were 9.9 ± 2.4, 10.6 ± 2.6 and 11.4 ± 0.7; the mean MSHQejD scores were 3.1 ± 0.9, 3.6 ± 0.7 and 4.0 ± 0.7, respectively. The mean MSHQ123 and mean MSHQejD scores were significantly increased at the first and third months of treatment compared to pretreatment values (*p* < .05). The mean EHS [25] scores at pretreatment and at the first and third month of treatment were 3.3 ± 1.1, 3.6 ± 0.7 and 3.7 ± 0.7, respectively. The mean EHS scores at the first and third month of treatment were significantly increased compared to the mean pretreatment score (*p* = .001).

The mean BDI scores at pretreatment and at the first- and third-month treatment periods were 2.9 ± 5.4, 2.5 ± 2.2 and 2.9 ± 2.3, respectively, but there was no statistically significant difference (*p* > .05). Four of the patients had borderline clinical depression (13.3%), and one had moderate depression (3.3%) throughout the treatment period. The mean pretreatment, and the first- and third-month treatment HARS scores were 19.3 ± 4.2, 12.5 ± 3.1 and 10.5 ± 2.6, respectively. The first month and third month of treatment mean HARS scores were statistically decreased compared to the mean pretreatment score (*p* = .001) (Table 3). The quality-of-life score improved at the end of the first month (*p* = .04) and third month (*p* = .02) of treatment.

## Discussion

Prior to the phosphodiesterase type-5 (PDE5) inhibitor era, ED treatment consisted of conservative options, including dialysis, correction of anemia using erythropoietin, testosterone supplementation, vacuum devices, penile or intraurethral vasoactive injections or surgical procedures, such as penile prosthesis and vascular surgery [26]. Although tadalafil is rapidly absorbed with a median 2 h of *T*_max_ in healthy men, and metabolism is completed in the liver, almost 36% of an oral dose is excreted in the urine [27]. To date, only a limited number of studies have investigated the effects of low-dose tadalafil in people with ESRD and on HD, although numerous studies have reported the effects of different sildenafil doses in such individuals. Chen et al. reported that sildenafil therapy increased the IIEF score by a minimum of 10 points in 80% of patients [28]. while Jacques et al. found that only two of 15 patients (13.3%) were satisfied with different doses of sildenafil treatment [29]. Our study revealed that low-dose tadalafil administration twice-weekly for 3 weeks resulted in significant improvement in the total mean IIEF scores and ejaculatory function scores, irrespective of etiology. Of the 30 patients, 11 (36.6%) had no ED at the end of the third month of therapy.

Many studies have reported that patients have discontinued tadalafil treatment due to adverse effects, or the lack of drug efficacy. Although none of the patients in the present study stopped the therapy due to minor side effects, 28 (93.3%) stated that they would not be able to continue the therapy for a long time because tadalafil was not covered by their insurance. It has previously been reported that diabetes mellitus and hypertension are the comorbidities most commonly associated with ED in HD patients [30]. Although the use of more than one antihypertensive agent has been reported in the treatment of hypertension for greater risk of ED, only six of the 30 patients (20%) were taking an alpha blocker for hypertension [31]. In the present study, we found no relationship between sexual dysfunction and hormonal and metabolic states, which was in accordance with previous studies [32].

Of the patients in the present study, 26 were hypoandrogenic due to abovementioned reasons, in accordance with previous studies, but none were hyperprolactinemic [33]. Another factor that alters erectile function is endothelial dysfunction [34]. It is expected that the more endothelial dysfunction occurs, the more likely it is that NO production decreases. Kensinger et al. showed that the increased amount of fibroblast growth factor-23 that is observed in ESRF was associated with endothelial dysfunction 2 years after renal transplantation. That suggested that the progressive endothelial dysfunction process continues in people with ESRD [35]. It has been shown that tadalafil decreases arterial stiffness and improves left ventricular diastolic function, even in patients with no cardiovascular or atherosclerotic diseases [36]. In contrast with this report, Pelliccione et al. claimed that tadalafil had only a modest effect on endothelial recovery [37]. Our results suggested that optimum effort must be made to guard against other cardiovascular risk factors, such as hypertension and hyperlipidemia, which promote endothelial dysfunction, in addition to using tadalafil treatment in patients with ESRD.

A limited number of previous studies have investigated ejaculatory disorders in ESRD. In those studies, ejaculation disorders were reported in 28–51.5% of HD patients [38–42]. Ejaculatory dysfunction is a common comorbidity with the prevalence of 25–40% [41]. In a review by Paduch et al., 57.8% of the 12,130 participants had abnormal ejaculation, and severity of ED was associated with poorer ejaculatory function [42]. Corona et al. reported that testosterone had a central and a peripheral role in ejaculatory reflex; according to widely accepted opinion, ejaculatory dysfunction might be mitigated following primary treatment of ED with PDE5 inhibitors [43]. The results of the present study revealed that tadalafil improved the mean MSHQ123 and MSHQ EjD scores throughout the follow-up period, which supports that hypothesis.

Psychogenic causes are another etiological reason in ESRD patients compared to age-matched counterparts without renal failure [44]. According to the World Health Organization, the prevalence of depression is approximately 16% [45]. Chronic illnesses contribute to this high prevalence, because psychosocial status is negatively affected, and patients are at risk of physical restrictions throughout their life. It has been shown that ED and stress negatively interact with each other and could lead to psychosocial problems [46]. Melnik et al. reported that psychotherapy plus PDE-5 inhibition resulted in significant improvement compared to PDE-5 inhibition alone, [47] while Baek et al. reported that tadalafil decreased depressive symptoms by affecting apoptotic neuronal cell death suppression in rats [48].

In the present study, none of the patients showed depression according, to the BDI which was stable throughout the 3-month period. In a review, Chilcot et al. reported that the prevalence of depression was 8.1–71.4% in different studies [49]. However, the mean HARS score decreased by 45.6% with tadalafil treatment. In addition to erectile improvement, lack of alterations in testosterone, hemoglobin, and lipid profile suggested that the positive impact of tadalafil on anxiety was associated with higher sexual quality and satisfaction [50,51].

### Limitations of this study

The short duration of therapy due to treatment costs, the low number of patients studied, and an absence of evaluation of the patients' partners.

## Conclusions

Tadalafil therapy is an effective therapeutic option in people with ESRD who undergo HD. Tadalafil 5 mg twice weekly not only improved ED, but also improved ejaculatory function, with acceptable adverse effects. Correctable conditions, such as hypertension and hyperlipidemia, which promote endothelial dysfunction, may influence treatment success rate, and a systematic approach is obligatory for improved outcomes.
